# Use of Highly Dispersed Mixed Metal Hydroxide Gel Compared to Bentonite Based Gel for Application in Drilling Fluid under Ultra-High Temperatures

**DOI:** 10.3390/gels9070513

**Published:** 2023-06-25

**Authors:** Bowen Zhang, Qingchen Wang, Xiaofeng Chang, Weichao Du, Fan Zhang, Michal Kuruc, Michal Slaný, Gang Chen

**Affiliations:** 1 Shaanxi Province Key Laboratory of Environmental Pollution Control and Reservoir Protection Technology of Oilfields, Xi’an Shiyou University, Xi’an 710065, China; 2 Shaanxi University Engineering Research Center of Oil and Gas Field Chemistry, Xi’an Shiyou University, Xi’an 710065, China; 3 CNPC Chuanqing Drilling Engineering Company Ltd., Xi’an 710018, China; 4 Department of Materials Engineering and Physics, Faculty of Civil Engineering, Slovak University of Technology, Radlinského 11, 810 05 Bratislava, Slovakia; 5 Institute of Inorganic Chemistry, Slovak Academy of Sciences, Dúbravská cesta 9, 845 36 Bratislava, Slovakia; 6 Institute of Construction and Architecture, Slovak Academy of Sciences, Dúbravská cesta 9, 845 03 Bratislava, Slovakia

**Keywords:** drilling fluid, mixed metal hydroxide-like compounds, low viscosity, high yield point

## Abstract

In order to solve the problem of poor dispersion and stability of mixed metal hydroxide (MMH), a kind of mixed metal hydroxide-like compound (MMHlc) gel was synthesized for use as the base mud in drilling fluid instead of bentonite gel. Na_2_CO_3_, Na_2_SiO_3_, and C_17_H_33_CO_2_Na were used as precipitants to form MMHlc with larger interlayer spacing and smaller particle size. MMHlc was synthesized by the coprecipitation method at 25 °C with a metal molar ratio of Mg:Al:Fe = 3:1:1. The performance evaluation of the treated drilling fluid showed that MMHlc (S2) synthesized using Na_2_SiO_3_ as the precipitant had the characteristics of low viscosity, low filtration, and a high dynamic plastic ratio at 25 °C, which fully met the requirements of oil field application, and it maintained its excellent properties after being aged at 250 °C for 16 h. Linear expansion and rolling recovery experiments showed that the S2 sample had excellent rheological properties and good inhibition. X-ray diffraction and FT-IR experiments showed that S2 had the most complete crystal structure, its interlayer distance was large, and its ion exchange capacity was strong. The thermogravimetric experiment showed that the S2 crystal was stable and the temperature resistance of the crystal could reach 340 °C. Zeta potential, particle size analysis, SEM, and TEM results showed that S2 is a nanomaterial with a complete morphology and uniform distribution. The drilling fluid of this formula had the characteristics of low viscosity, low filtration loss, and a high dynamic plastic ratio, and it met the conditions for oil field application. Considering these results, the new MMH prepared by our research institute is a drilling fluid material that can be used at ultra-high temperatures and can provide important support for drilling ultra-deep wells.

## 1. Introduction

Mixed metal hydroxide (MMH) is a kind of gel, also known as mixed metal carboxyl salt, which is additionally known as positive gel in oil and gas fields in China [1]. In the 1980s, Burba [2] and others first proposed the use of MMH as a drilling fluid inhibitor. The combination of MMH and drilling fluid mud forms a drilling fluid system with strong inhibition. It forms a layered or a three-dimensional network structure composed of octahedral stacking layers [3]. In the crystal structure of MMH, the layered structure is formed by the interactions of hydrogen bonds and ionic bonds, and the gaps between the layers can accommodate water molecules and other molecules. This layered structure can be stable at high temperature, has good thermal stability, and can resist physical and chemical changes in a high-temperature environment. MMH also has a strong ion exchange capacity. The metal ions in the mixed metal hydroxide can exchange with other ions (such as potassium ions, sodium ions, etc.), so that the MMH surface has a strong positive charge. In the clay layer, it is tightly adsorbed on the layer’s surface, drawing the layered structure of the clay to reduce the entry of water molecules. MMH has a large specific surface area and certain pore structure, which can adsorb and remove organic substances and heavy metal ions in drilling fluid to reduce the content of pollutants [4].

At present, most of the mixed metal hydroxides are composed of magnesium and aluminum. Bowen Zhang et al. [5] studied the effects of the proportions of magnesium and aluminum. With an increase in the proportions of magnesium and aluminum, the performance of the MMH gradually became better, and the MMH remained stable after aging at 180 °C for 16 h. Bowen Zhang et al. [6] studied multi-mixed metal hydroxides (M-MMHs), which generally have more complex crystal structures because they contain multiple metal ions and oxide ions. These ions can be arranged in different positions, forming a variety of structures, resulting in more complex physical and chemical properties. The M-MMHs have high stability; they are still stable after being aged at 200 °C for 16 h and can maintain stability in a wide range of pH values. In contrast, binary metal hydroxides are easily affected by the environment and lose stability. M-MMHs have better physical and chemical properties, such as higher thermal stability, chemical stability, and oxidation resistance, and can better adapt to drilling operations under high temperature, high pressure, and extreme environments [7]. An evaluation of M-MMH drilling fluid performance showed that M-MMH has unique rheological properties, with low viscosity, high shear force, and high dynamic plastic ratio, and meets the requirements of drilling fluid for deep wells and horizontal drilling. M-MMH is a solid-free drilling fluid that will not block the cracks of oil and gas reservoirs. At the same time, the temperature resistance limit of bentonite is 200 °C, which restricts improvements in the temperature resistance of water-based drilling fluid [8]; this has inspired attempts to use M-MMH instead of bentonite in drilling fluid base mud.

Bentonite is mainly composed of montmorillonite, which is the most widely used water-based drilling fluid base mud in oil fields [9]. Montmorillonite is composed of two silicon–oxygen tetrahedra and one aluminum–oxygen octahedron, which has good hydration expansion ability. The interlayer distance of bentonite in water increases from 0.96 nm to 4.0 nm. Its good expansion causes the mud to constitute a relatively stable gel [10]. However, the temperature limit of bentonite is between 150 °C and 200 °C, so bentonite becomes an important factor limiting the application temperature of water-based drilling fluid. The temperature resistance of M-MMH can reach 200–250 °C, and MMH has unique rheological properties, low viscosity, and high shear force [11]. Wan-Guo et al. [12] found that Mg-Al MMH has good stability and fluid loss reduction under high temperature and high pressure and can effectively reduce the viscosity of drilling fluid. M-MMH has good colloidal properties, can form a solid wall film, can effectively control wellbore stability, and can reduce bit wear and wellbore collapse. Other effects include reducing the width of the contact zone between the borehole wall and the stratum, improving the recovery rate of the drilling fluid, reducing the loss of the drilling fluid, and reducing the risk of environmental pollution [13].

However, because the interlayer distance of M-MMH is too small, ion exchange does not easily occur and water molecules do not easily enter the interlayer; thus, the dispersion of M-MMH is poor and its hydration expansion capacity is not strong, so it does not form a relatively stable gel. Meanwhile, the uneven particle size of M-MMH also leads to the problem of poor dispersion, which leads to the problem of excessive filtration of M-MMH as the base mud of drilling fluid [14]. To solve these problems, the first step is to select a precipitant with a large anion radius and strong exchangeability to increase the distance between layers and the exchange capacity. The second step is to select a weak alkaline precipitant to slow down the precipitation process, and the metal ions should fully react with the precipitant to generate MMHlc with smaller particles to avoid agglomeration. Na_2_CO_3_, Na_2_SiO_3_, and C_17_H_33_CO_2_Na were selected as alkaline precipitants to synthesize MMHlc. Finally, an anionic surfactant was selected to improve the overall performance.

## 2. Results and Discussion

### 2.1. Synthesis of MMHlc

**Figure 1:**
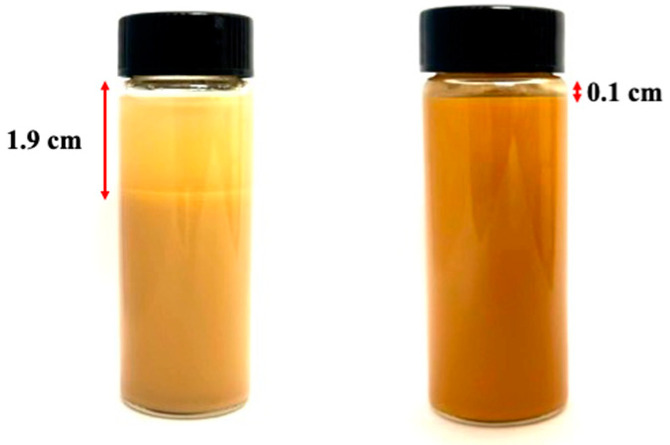
Samples left to stand for 24 h after aging at 250 °C (**left**: 4% base mud, **right**: 4% S2).

The surfaces of ions and water molecules in MMHlc have electrostatic charge, which can adsorb ions or molecules with opposite charges to form an electric double layer. For instance, when MMHlc is in water, the positively charged metal ions on the surface can adsorb negatively charged water molecules or other anions, thereby forming an electrical double layer [15]. This results in the formation of stable gels in water, so the reaction can be slowed down by adding a weakly alkaline solution, thereby reducing the particle size of the mixed metal hydroxide. It is also possible to achieve dispersion stability by increasing the ion exchange property [16]. It can be seen from Figure 1 that the gels of the three samples were relatively stable, with fine particles and good fluidity, indicating that MMHlc synthesized from a weak base solution has smaller particles. The rheological properties of the drilling fluid made using the three samples at 25 and 250 °C were analyzed, and the results are shown in Table 1.

It can be seen from Table 1 that the 4% samples at 25 °C showed a good dynamic plastic ratio and, at the same time, solved the shortcoming of excessive filtration loss of M-MMH as the base mud. At 25 °C, the filtration loss of 4% S1 was 13.5, and the filtration loss of 4% S2 was 16.7 mL, which was decreased by 31.8 mL compared with the 48.5 mL filtration loss of the base mud. The high dynamic plastic ratio of MMHlc was retained as the filtration loss improved. The dynamic plastic ratio of 4% S2 was 2.14 at 25 °C. MMHlc showed the characteristics of low viscosity, high shear force, and high dynamic plastic ratio at 25 °C. After aging at 250 °C for 16 h, the three-dimensional structure of the lime clay base mud was completely destroyed, the filtration loss was as high as 150 mL, and the apparent viscosity decreased from 5.05 mPa·s to 2.75 mPa·s, indicating that the bentonite gel was completely broken at 250 °C. However, MMHlc maintained good stability, among which 4% S2 retained a good state; its filtration loss was the best among the three samples at 45.6 mL, and its gel stability was the best. After standing for 24 h, the delamination of 4% S2 was obviously less than that of 4% base mud, as shown in Figure 1.

It can be seen from Figure 1 that the base mud had obvious stratification after being left to stand for 24 h, but the stratification of 4% S2 was not obvious, indicating that S2 remained stable at 250 °C; this could be due to the high temperature reducing the particle size of S2.

### 2.2. XRD

**Figure 2:**
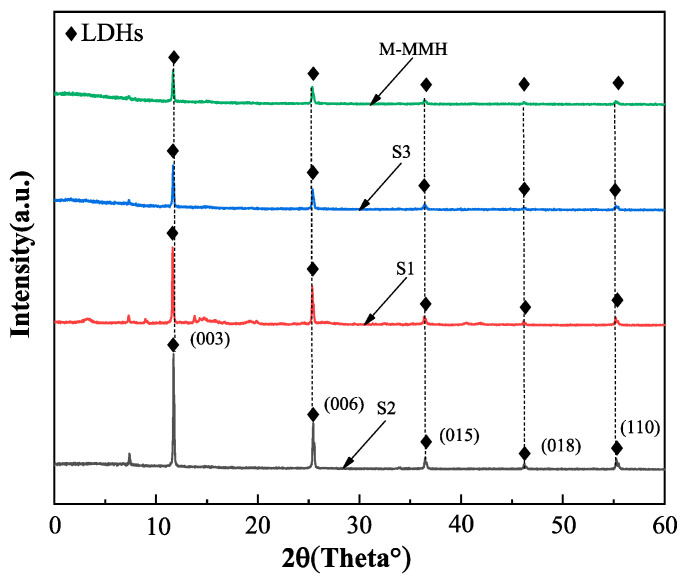
XRD experimental results of MMHlc samples.

We took small samples of S1, S2, and S3, passed them through a 100-mesh sieve, and conducted XRD testing. The results are shown in Figure 2. It can be seen from Figure 2 that MMHlc and M-MMH had similar crystal structures, belonging to hexagonal crystals, but there was a strong reflection peak at 003; the reflection peak of S2 at 003 was the strongest, indicating that the crystal of S2 was most similar to a hexagonal system and had a high degree of crystallization [17,18]. The crystal spacings of S1, S2, and S3 were calculated according to the Bragg equation. For S1, d(003) = 1.711 nm; for S2, d(003) = 2.786 nm; and for S3, d(003) = 1.928 nm. This indicates that the addition of silicate significantly increased the interlayer structure of the crystal. Compared with those of oleate and carbonate, the anion radius of silicate is larger and the charge density is lower, which leads to weak electrostatic force between the layers; the larger anion radius also supports interlayer spacing in the MMHlc. To sum up, S2 has a relatively complete static layer structure to ensure better ion exchange capacity, and a larger interlayer distance can accommodate more water, so S2′s hydration expansion in water is significantly better than that of S1 and S3.

### 2.3. FTIR Analysis

**Figure 3:**
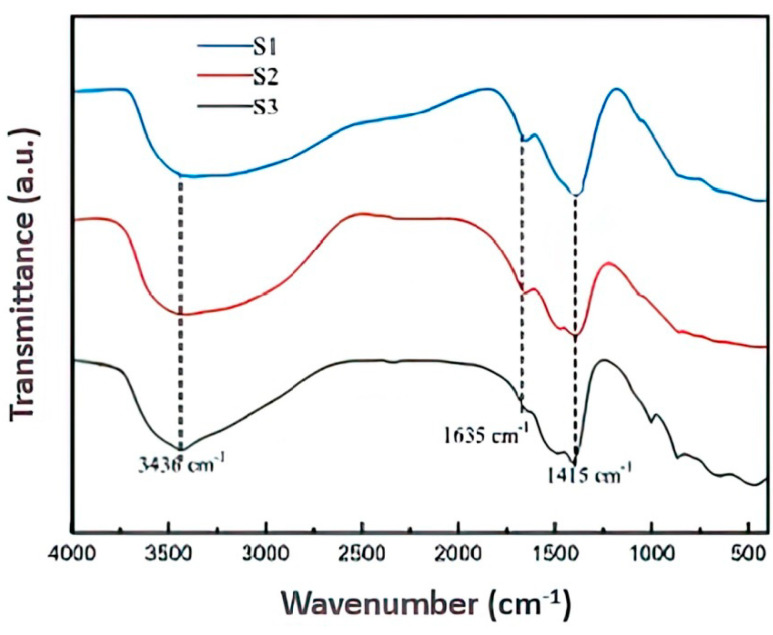
FTIR spectra of S1, S2, and S3.

FTIR experiments were performed on MMHlc in a gel state, and the results are shown in Figure 3. It can be seen from Figure 3 that the infrared spectra of the three MMHlc samples were not much different. Relatively broad absorption bands at 3436 cm^−1^ and 1635 cm^−1^ correspond to the OH stretching and bending vibrations of water molecules present in the montmorillonite, respectively. These peaks also represent the water molecules in the hydrotalcite, and the absorption bands occurring in samples S2 and S3 most obviously indicated that the hydration of S2 and S3 is much higher, which was consistent with the evaluation results of drilling fluid performance. The absorption band at 1415 cm^−1^ could be summarized as Si–O–Si, which is the supporting skeleton of the sample structure and forms an infinitely extended layered structure by coplanar connection. At the same time, the polarity of the Si–O–Si bond causes the hydrotalcite to have good hydrophilicity. In the infrared spectra, several small peaks are visible between 500 cm^−1^ and 1000 cm^−1,^ which could be summarized as Si–O–M. This represents the metal ions Mg^2+^, Al^3+^, and Fe^3+^, which play a charge-balancing role in the crystal structure. At the same time, Si–O–M bonds can exchange ions with cations in other anionic surfactants to form an adsorption layer, thereby enhancing the performance of MMHlc as an adsorbent [19,20]. To sum up, MMHlc and bentonite have similar infrared spectral structures, strong ion exchange capacity between layers, and more water between the layers of S2, which is consistent with the XRD experimental results and proves that S2 has good hydration and dispersion capacity.

### 2.4. TGA

**Figure 4:**
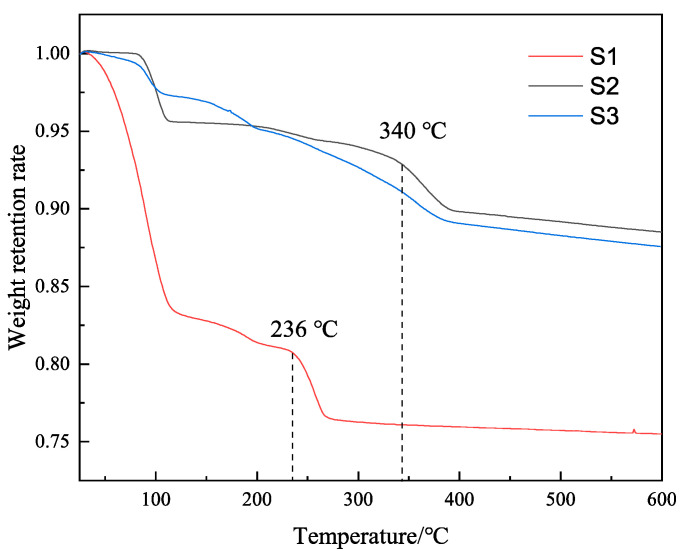
TG curves of the three MMHlc samples.

We centrifuged the MMHlc samples, took a small amount of lower sediment, and used it for TGA after drying. Figure 4 shows the TGA results of samples S1, S2, and S3.

It can be seen from Figure 4 that the first weight loss occurred at 100 °C. This was due to the water molecules between the sample layers. The weight loss rate of S1 was the largest, indicating that the water molecules between the layers of S1 were the most numerous at 25 °C, which was similar to the rheology of the drilling fluid, and the hydration degree of S1 at 25 °C was relatively high. However, with increasing temperature, the second mass collapse of S1 occurred at 236 °C, which indicated that the crystal of S1 was destroyed at 236 °C. It can be seen from the results for S2 that its weight remained stable at 25–100 °C and at 100–340 °C, indicating that the crystal of S2 was always in a stable state. However, the weight of S3 continuously decreased during the process at 25–350 °C, indicating that the crystal structure of S3 was unstable and in a state of continuous collapse. S2 showed not only good ion exchange but also good temperature resistance and crystal stability, which was consistent with the results of the drilling fluid performance evaluation.

### 2.5. SEM and TEM

**Figure 5:**
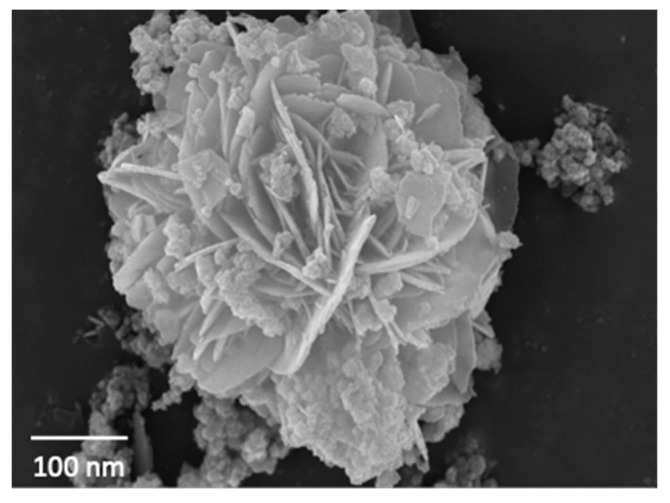
SEM image of sample S2.

**Figure 6:**
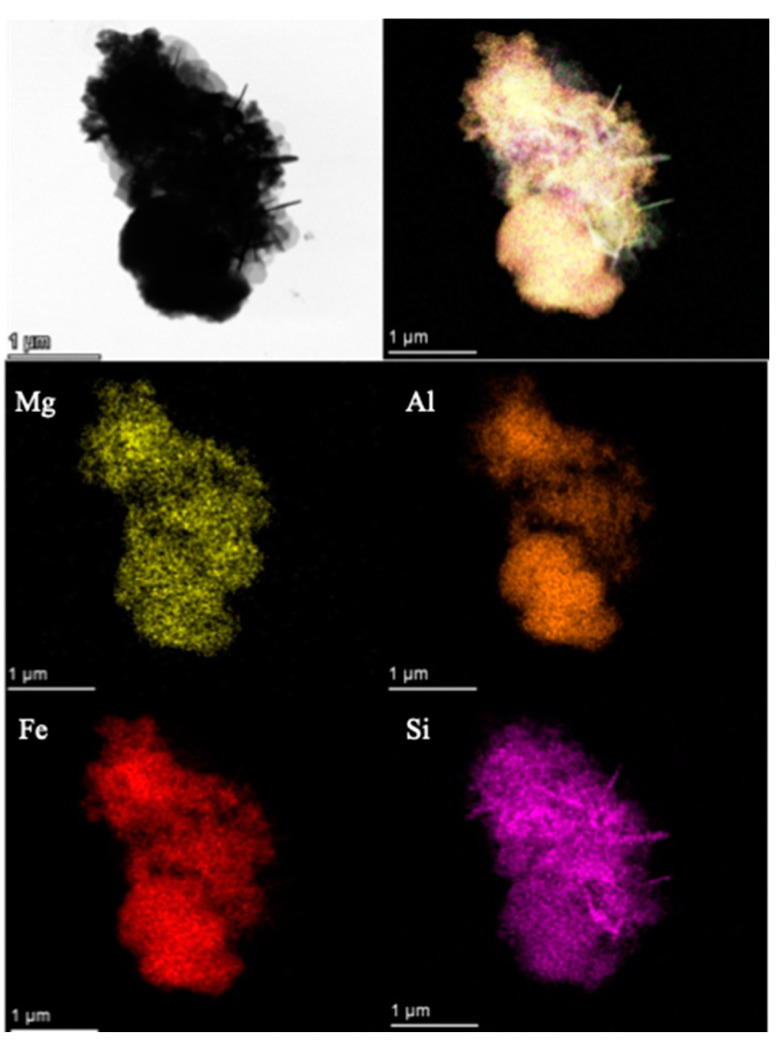
Element distribution of sample S2.

**Figure 7:**
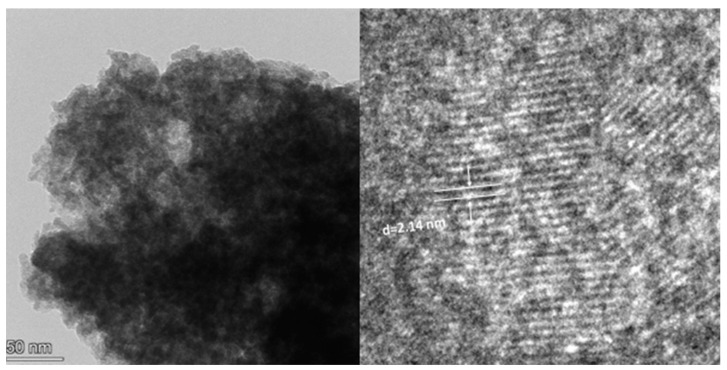
TEM image of S2.

The hexagonal structure of S2 was evident, with dense lamellar structures on the layers. The sample was evenly distributed. To further understand the composition structure of the sample, the content and type of various elements in the hydrotalcite were analyzed, and the energy spectrum analysis results of the sample are shown in Figure 5 and Figure 6. It can be seen from Figure 5 that S2 exhibited an obvious hexagonal layered structure with a uniform distribution and particle size between 200 and 300 nm, showing a smaller particle size and more complete crystal structure. It can be seen from Figure 6 that the elements of Fe, Mg, Al, and Si in S2 were uniformly distributed, indicating that the synthesized crystal was relatively complete and was not separate precipitates of Fe, Mg, Al, and Si; this conformed to the XRD experimental results. The more complete crystal structure represents better stability, which further explains the stability of the S2 samples. In order to further observe the microstructure of S2, a TEM experiment was performed, and the results are shown in Figure 7.

**Figure 8:**
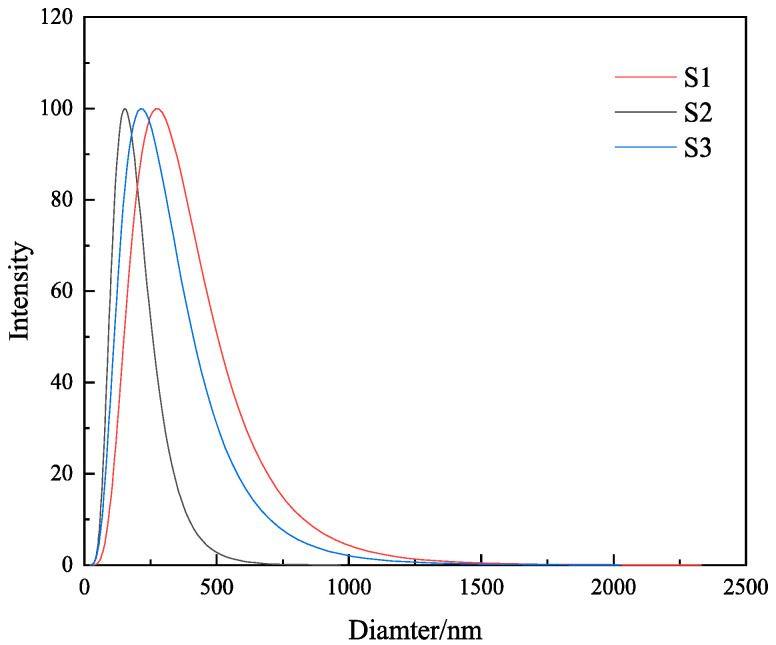
Particle size analysis of the three MMHlc samples.

It can be seen from Figure 8 that the morphology of the S2 sample comprised hexagonal particles, and the samples were arranged in a sheet structure. Compared with that of M-MMH, the particle size of the sample was obviously smaller [21], which proves that the addition of silicate radicals reduced the particle size of the sample. The median particle size of the sample was 178.12 nm, which is similar to the result measured in the Zeta potential experiment, further indicating that MMHlc is a nano-scale material; the smaller particle size brings better dispersion and ensures the inhibition of the sample. The interlayer distance of 2.14 nm was measured by TEM, which proved the XRD result of an obviously increased interlayer distance.

### 2.6. Analysis of Zeta Potential

Zeta potential tests were performed on the three samples at 25 °C, and the results are shown in Table 2 As shown in Table 2. the Zeta potential values of 4% S1, S2, and S3 samples at 25 °C were 41.23 mV, 52.16 mV, and 39.13 mV, respectively. The Zeta potential value of sample S2 was the highest, which proved that the surface of the particles had greater charge. The mutual repulsion between particles was thus enhanced, and the stability was also enhanced. On the contrary, when the Zeta potential is low, the surface of the particles has less charge, the mutual attraction between the particles is enhanced, and the stability is reduced. Therefore, the gel stability of S2 was the best, which is in accordance with the success of S2 in previous experiments.

### 2.7. Particle Size Measurement

Particle size analysis was performed on the gels of the three samples S1, S2, and S3, and the results are shown in Figure 8 and Table 3. It is clearly visible from Table 3 and Figure 8 that the mean particle sizes of 4% S1, S2, and S3 were 345.2 nm, 178.5 nm, and 432.1 nm, respectively, and the median particle sizes of 4% S1, S2, and S3 were 378.1 nm, 165.4 nm, and 446.1 nm, respectively. The particle size of S2 was the smallest, which proved that the MMHlc synthesized using Na_2_SiO_3_ was a nano-scale product. The small particle size of the sample proves that S2 had a large specific surface area, and the smaller particle size represents a better dispersion; the sample could thus be more uniformly dispersed in water, avoiding agglomeration precipitation, and filtration loss was consequently reduced. The particle size distribution of S2 was relatively uniform, which meant that the surface of S2 was smoother and more stable, and physical and chemical reactions could not easily occur, which is conducive to long-term storage.

### 2.8. Linear Expansion Experiment on MMHlc

**Figure 9:**
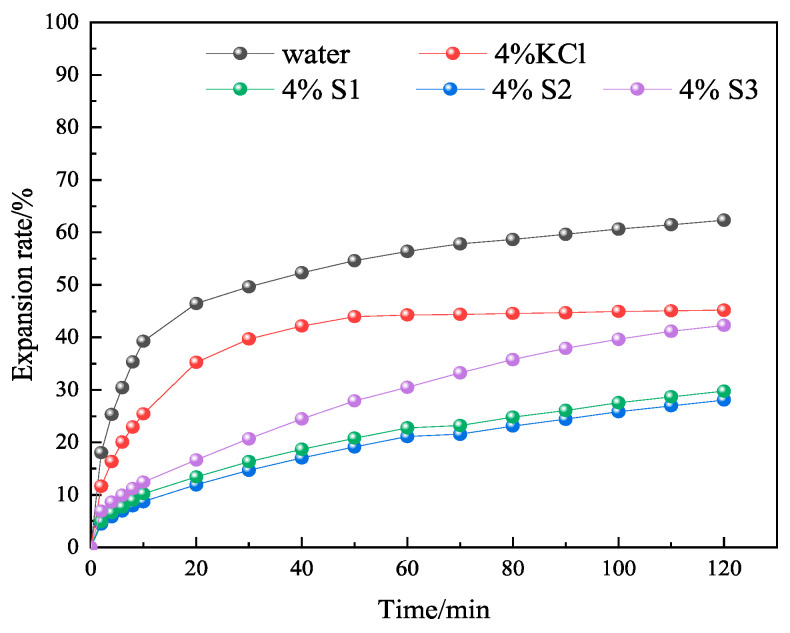
Experimental results of linear expansion of S1, S2, and S3 at 25 °C.

**Figure 10:**
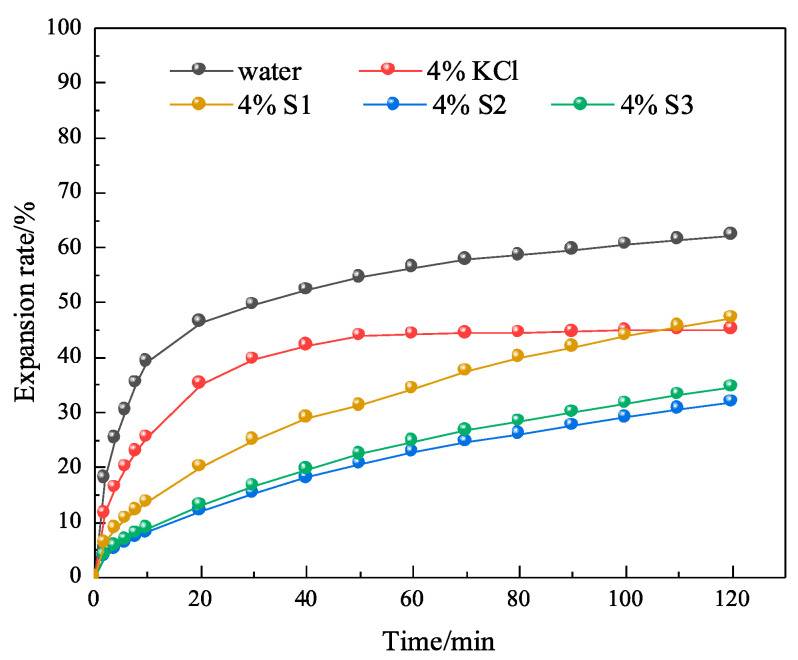
Experimental results of linear expansion of S1, S2, and S3 at 250 °C.

In order to further investigate whether MMHlc had an effect on inhibition while improving the rheological properties, linear expansion tests were conducted using 4% S1, S2, and S3 gels aged at 25 and 250 °C for 16 h. The linear expansion tests were conducted at 25 °C, using clean water and 4% KCl solution as controls. The results are shown in Figure 9 and Figure 10.

From Figure 10 it’s possible to see that the linear expansion rate of bentonite in clean water at 25 °C was 63.12% at 120 min, and the linear expansion rate of 4% KCl solution at 25 °C was 46.16% at 120 min. At 25 °C, the 120 min linear expansion rates of 4% S1, 4% S2, and 4% S3 were 42.17%, 27.94%, and 29.75%, respectively. The linear expansion rate of S2 was similar to that of S3, which proved that they were similar in their inhibition. The linear expansion rate of S2 was 33% higher than that of S1, which proved that although S1 had the lowest filtration loss at 25 °C, its inhibition was not good, so the comprehensive performance of S2 was the best.

**Figure 11:**
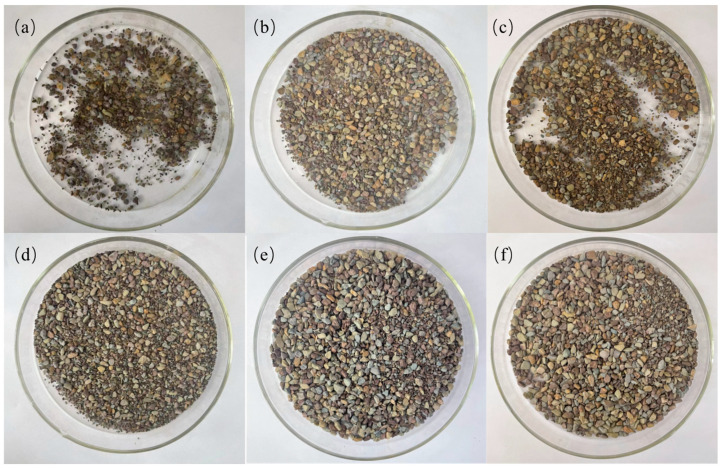
Results of the second rolling recovery experiment (**a**) water, (**b**) 7% KCl, (**c**) 4% base mud, (**d**) 4% S1, (**e**) 4% S2, (**f**) 4% S3.

After 4% S1, S2, and S3 were aged at 250 °C for 16 h in a reactor, their inhibition was evaluated, as shown in Figure 11. After high-temperature aging, the linear expansion rates of 4% S1, 4% S2, and 4% S3 at 120 min were 47.86%, 31.84%, and 35.65%, respectively. The results showed that the S2 sample retained its stability after aging at 250 °C for 16 h, which was consistent with the performance evaluation of drilling fluid. The results showed that the crystal structure and three-dimensional network structure of S2 were not destroyed at 250 °C.

### 2.9. Experimental Analysis of Shale Rolling Recovery

For fragile rock formations, the rolling recovery test can intuitively reflect the state of drilling fluid and cuttings in the formation. We took 50 g of shale with a size of 10 mesh to 20 mesh, prepared 350 mL of MMHlc suspension, added it to the tank, and rolled it at 120 °C for 16 h. The results are shown in Table 4 and Figure 11.

From Figure 11 and Table 4 is visible that in the rolling recovery test at 120 °C, the first rolling recovery of clear water was only 9.8% and 23.8% in 7% KCl. The three MMHlc samples have superior inhibition, among which S2 performs the best. The first rolling recovery of shale in 4% S2 was 66.0%, and its second rolling recovery was 64.1%. The results of the experiments conducted in this study show that the recovery rates for the first and second cycles are similar, with good regularity. This indicates that these inhibitors have strong adsorption on shale.

### 2.10. Mechanism Discussion

**Figure 12:**
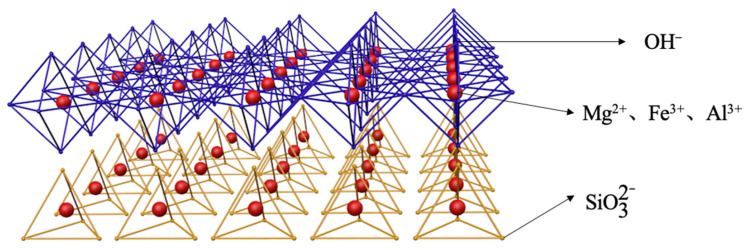
Crystal diagram of MMHlc.

MMH is a kind of regular hexagonal crystal, closely arranged, with a layered structure and is a kind of LDH. The composition of MMHlc crystals synthesized using sodium silicate is similar to that of MMH, which is generated by the iterative aggregation of mixed metal ions surrounded by multiple anionic groups, divided into a silicic acid layer and a hydroxide layer [22,23]. The silicic acid layer is a tetrahedral structure composed of silicate ions, which are connected in such a way that each silicon atom shares a vertex with three adjacent oxygen atoms, forming a three-dimensional network structure. In MMHlc, the silicic acid layer consists of four silicate ions forming a ring; the metal ions in the ring may be Mg^2+^, Fe^3+^, and Al^3+^, and a hexagon is formed around the metal ions. Meanwhile, sodium silicate can undergo a hydrolysis reaction in water to generate NaOH and H_2_SiO_3_, wherein NaOH can react with metal ions Mg^2+^, Fe^3+^, and Al^3+^ in the solution to form a hydroxide layer. The metal ions are surrounded by eight hydroxide ions to form an octahedral structure, and the silicic acid layer and the hydroxide layer are tightly connected through oxygen ions and hydrogen bonds. These two layers are alternately arranged to form the structure of MMHlc, which is shown in Figure 12.

The crystal structure of MMHlc synthesized from sodium silicate is similar to that of montmorillonite. Montmorillonite is composed of two silicon–oxygen tetrahedra and one layer of aluminum–oxygen octahedron, while the structure of the S2 sample is a layered arrangement of tetrahedra and octahedra, which leads to more layers of S2. Meanwhile, the anion radius of interlayer silicate radicals is larger and the charge density is lower, which leads to weak electrostatic force between the layers, resulting in larger interlayer spacing. Meanwhile, the larger anion radius also supports the interlayer spacing of the MMHlc. It more easily absorbs water and expands to form a stable gel. The unique rheological behavior of sample S2 is due to its higher positive surface charge and unique layered structure; the synthesized particle size is smaller, which leads to a better specific surface area of hydrotalcite. There are a large number of ion exchange sites between the layers. When the sample is suspended in water, the interlayer ions with negative charges on the surface adsorb the surrounding cations and anions, forming a stable electrical double layer. When an external force acts on the suspension of the sample, the electric double layer can form a structure similar to an electric double layer capacitor, so that the rheological properties of the suspension of the S2 sample are controlled by the electric field and show a special rheological behavior. The MMHlc shows higher viscosity at low bit speed and lower viscosity at high bit speed due to the weakening of its interlayer electrostatic strength, which results in a higher dynamic plastic ratio and lower viscosity.

## 3. Conclusions

Among the three samples S1, S2, and S3, the filtration loss of 4% S2 was 16.7 mL, which was decreased by 31.8 mL compared with the 48.5 mL filtration loss of the base mud and 120.6 mL of MMH. The high dynamic plastic ratio of MMH was retained as the filtration loss improved, and the dynamic plastic ratio of 4% S2 was 2.14 at 25 °C. After aging at 250 °C for 16 h, the three-dimensional structure of the base mud was completely destroyed, and its filtration loss was as high as 150 mL. However, 4% S2 remained in good condition, and its filtration loss was the best among the three MMHlc samples (45.6 mL). MMHlc showed low viscosity, high shear force, and a high dynamic plastic ratio at 25 and 250 °C. The linear expansion and rolling recovery experiments showed that the linear expansion rate of 4% S2 was 27.94% after 120 min at 25 °C, and the linear expansion rate of 4% S2 was 31.84% after aging at 250 °C for 16 h. In the rolling recovery experiment, the first rolling recovery rate of 4% S2 was 66.04%, and the second rolling recovery rate was 64.12%. The addition of silicate significantly increased the interlayer structure of the crystal. Compared with those of oleate and carbonate, the anion radius of silicate is larger and the charge density is lower, which leads to weak electrostatic force between the layers. The larger anion radius also supports the interlayer spacing of the MMHlc, thus accommodating more water. S2 had more water between its layers, which was consistent with the X-ray diffraction results. This proved that S2 had good hydration and dispersion ability. In conclusion, the MMHlc synthesized using Na_2_SiO_3_ as the precipitant had the best performance, displaying properties such as high temperature resistance, environmental protection, low filtration loss, low viscosity, and a high dynamic plastic ratio.

## 4. Materials and Methods

### 4.1. Materials

Ca bentonite (technical grade; mineralogical composition: 60% montmorillonite, 25% quartz, 15% feldspar) was purchased from Xi’an Chanqing Chemical Co., Ltd. (Shannxi, Xian, China). MgCl_2_, AlCl_3_, FeCl_3_, and NaOH (technical grade) were purchased from Tianjin Shengao Chemical Reagent Co., Ltd. (Shenggao Inc., Tianjin, China).

### 4.2. Synthesis of MMHlc

Based on the research content of Zhang Bowen [6], the molar ratio of raw materials of an M-MMH is Mg:Al:Fe = 3:1:1, and Na_2_CO_3_, Na_2_SiO_3_, and C_17_H_33_CO_2_Na were used as precipitants instead of NaOH to improve the dispersion of the samples. The process is as follows: add MgCl_2_, AlCl_3_, and FeCl_3_ into 300 mL of water at 25 °C and stir well. Add 100 mL of water into the alkaline precipitant, add 1% (mass ratio) of isopropanol into Na_2_SiO_3_ and C_17_H_33_CO_2_Na for solubilization, and then add this slowly, dropwise, into the metal salt solution while stirring. Stir for a further 1.5 h to obtain the final sample. After obtaining the sample, do not dry it, but directly use it as a product to evaluate its rheology, Zeta potential, particle size, infrared spectra, and other characterizations. Centrifuge a portion of the sample and take the lower sample. Dry the obtained sample at 95 °C for 36 h to obtain a dried sample, which can then be ground into powder and passed through a 100-mesh sieve to facilitate subsequent X-ray diffraction experiments, infrared spectroscopy, and thermogravimetric experiments. MMHlc can undergo a hydrolysis reaction in water; its reaction equation is shown in Formula 1:

M_x_N_y_ + zH_2_O = xM^z+^(aq)+yN^z−^(aq) (1)

**Figure 13:**
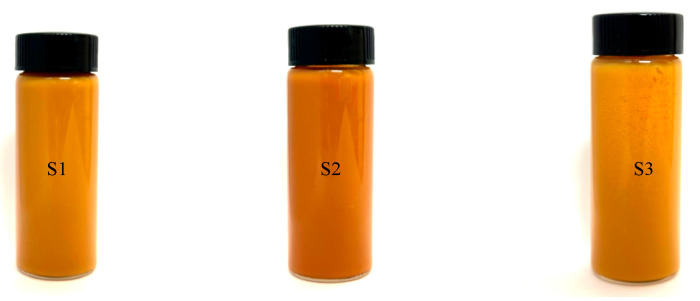
Sample morphology at 25 °C.

Here, M^z+^ represents Mg^2+^, Al^3+^, or Fe^3+^ metal ions, and N^z−^ represents anions such as ${\mathrm{CO}}_{3}^{2-},{\mathrm{SiO}}_{3}^{2-},{\mathrm{RCOO}}^{-}$, and the like. The product synthesis catalogue is shown in Table 5, and the sample morphology is shown in Figure 13.

### 4.3. X-ray Diffraction

The samples were analyzed using a D8ADVAHCL X-ray diffractometer (Bruker, Saarbrücken Municipality, Germany). A Cu target, ceramic X-ray tube, tube current of 40 mA, tube voltage of 40 kV, step size of 0.02°, and scanning range of 0~60° (2θ) were used for the measurements. The variation in the interlayer spacing of the sodium bentonite under different conditions was calculated using the Bragg equation (nλ = 2dsinθ) [24].

### 4.4. FTIR Spectroscopy

The infrared spectra were collected using a Nicolet 6700 Fourier transform infrared (FTIR) spectrometer from Thermo Scientific™ (Waltham, MA, USA). An IR source, KBr beam splitter, and DTGS detector were used for the mid-IR measurements (4000–400 cm^−1^) [25].

### 4.5. TGA

TGA was performed using a TGA/DSC1/1600 thermal analysis machine (Mettler Toledo Inc., Zurich, Switzerland) [26].

### 4.6. TEM and SEM

The surface morphology of samples was investigated using a digital imaging scanning electron microscope (model SU6600, serial no. HI-2102-0003, Hitachi, Japan). The sample was subjected to HRTEM analysis using a TEM Cs probe microscope (Waltham, MA, USA) after ion sputtering with gold for 45 s to explore the structural properties of the as-synthesized material [27].

### 4.7. Zeta and Particle Size Experiment

The Zeta potential of 4% M-MMH samples were measured using an Omni multi-angle particle size analyzer and a high-sensitivity Zeta potential analyzer (Booker Inc., Dallas, TX, USA) [28]. The experimental method was based on measuring the particle sizes of selected materials using a particle size analyzer: Laser—Partikel Sizer, Analysette 22, MicroTec plus, from company FRITSCH GmbH (Amberg, Germany) on powder and emulsion samples. Method of analysis was based on static light scattering (laser diffraction). Wet measurement method in full range from 0.08 to 2.5 μm was selected.

### 4.8. Drilling Fluid Performance Evaluation

The performance of the MMHlc drilling fluid, such as AV (apparent viscosity), PV (plastic viscosity), YP (yield point), FL (API filtration), was evaluated in accordance with oil and gas industry standard GB/T 16783.1-2006 [29].

### 4.9. Rolling Recovery Experiment

We referred to petroleum industry standard SY-T 5613-2000: test method for physical and chemical properties of shale [30].

### 4.10. Inhibitory Evaluation

The hydrated expansion of sodium bentonite was determined using a shale expander (NP01, Chuangmeng Ltd., Qingdao, China) according to the Chinese Petroleum and Natural Gas Industry Standards SY/T 5971-1994 and SY/T 6335-1997 [31].

## Figures and Tables

**Table 1:** Drilling fluid evaluation of MMHlc at 25 and 250 °C.

Temperature	Recipe	AV/ mPa·s	PV/ mPa·s	YP/ Pa	FL/ mL	Lubricity Factor	YP/PV
25 °C	4% base mud	5.05	4.80	0.25	48.5	0.23	0.05
	4% S1	6.05	2.00	4.05	13.5	0.25	2.03
	4% S2	6.60	2.10	4.50	16.7	0.27	2.14
	4% S3	7.75	3.00	4.75	22.1	0.22	1.58
250 °C	4% base mud	2.75	2.70	0.05	150.0	/	0.02
	4% S1	5.70	2.00	3.70	67.5	0.14	1.85
	4% S2	5.25	1.90	3.35	45.6	0.15	1.76
	4% S3	3.00	1.50	1.50	62.1	0.11	1.00

**Table 2:** Zeta potential values of different samples.

Name	Zeta Potential Value/mV
4% S1	41.23 (± 1.71)
4% S2	52.16 (± 2.12)
4% S3	39.13 (± 0.96)

**Table 3:** Particle size analysis of the three MMHlc samples.

Name	Average Particle Size/nm	Median Particle Size/nm
4% S1	345.2 (± 3.1)	378.1 (± 2.7)
4% S2	178.5 (± 4.6)	165.4 (± 3.3)
4% S3	432.1 (± 3.7)	446.1 (± 1.7)

**Table 4:** Results of the rolling recovery experiment.

Name	First Rolling Recovery	Second Rolling Recovery
Water	9.8% (± 1.2)	8.4% (± 1.0)
7% KCl	23.8% (± 2.2)	21.1% (± 0.7)
4% Base mud	15.6% (± 1.6)	14.4% (± 1.2)
4% S1	50.2% (± 4.1)	48.7% (± 2.7)
4% S2	66.0% (± 3.6)	64.1% (± 1.9)
4% S3	37.0% (± 2.7)	30.1% (± 2.1)

**Table 5:** Product synthesis catalog.

Name	n_Mg_:n_Al_:n_Fe_	Precipitant	Product Status
S1	3:1:1	Na_2_CO_3_	Gel
S2	3:1:1	Na_2_SiO_3_	Gel
S3	3:1:1	C_17_H_33_CO_2_Na	Gel

## Data Availability

Not applicable.
